# Color Stability of Conventional and Bulk-Fill Resin Composites in Health-Oriented Beverages

**DOI:** 10.3390/ma19173637

**Published:** 2026-08-27

**Authors:** Zeynep Hale Keles, Gorkem Sengez, Aliye Tugce Gurcan, Soner Sismanoglu

**Affiliations:** 1 Department of Restorative Dentistry, Faculty of Dentistry, Istanbul Atlas University, 34408 Istanbul, Türkiye; 2 Private Practice, 34000 Istanbul, Türkiye; gorkemsengun@gmail.com; 3 Department of Pediatric Dentistry, Faculty of Dentistry, Dokuz Eylul University, 35340 Izmir, Türkiye; aliyetugce.gurcan@deu.edu.tr; 4 Department of Restorative Dentistry, Faculty of Dentistry, Istanbul University-Cerrahpasa, 34098 Istanbul, Türkiye; soner.s@hotmail.com

**Keywords:** color stability, bulk-fill composite, resin composite, discoloration, detox beverages, spectrophotometry

## Abstract

Objective: To compare the color stability of a conventional nanofilled composite (Filtek Ultimate, FU) and a bulk-fill composite (Filtek Bulk Fill, FB) after immersion in health-oriented beverages. Materials and Methods: A total of 120 disc-shaped specimens (n = 10 per group) were prepared from the two composites and immersed for 12 days in distilled water (control), orange juice, green detox, red detox, pomegranate juice, or multivitamin water. Color coordinates (L*, a*, b*) were measured with a spectrophotometer at baseline and after immersion, and ΔL*, Δa*, Δb*, and ΔE were calculated using the CIELAB system. Data were analyzed using two-way ANOVA with Games-Howell and Welch’s *t*-tests (α = 0.05). Results: Material, immersion solution, and their interaction significantly affected all color parameters (*p* < 0.001). FB showed significantly higher ΔE than FU across all six solutions, including distilled water (*p* = 0.014). In FB, orange juice produced the highest color change (ΔE = 17.33 ± 1.61), followed by pomegranate juice (14.01 ± 0.33) and red detox (10.08 ± 0.72); in FU, ΔE ranged from 1.76 ± 0.76 (distilled water) to 5.16 ± 2.08 (orange juice). Orange juice caused a pronounced increase in lightness in FB (ΔL* = 16.12 ± 1.37), unlike the darkening produced by the other beverages. All beverage groups in both materials exceeded the acceptability threshold (ΔE = 2.7), whereas the distilled water controls of both materials remained below it. Conclusions: The bulk-fill composite was significantly more susceptible to discoloration than the nanofilled composite for all beverages. Health-oriented drinks, particularly orange, pomegranate, and red detox juices, produced clinically unacceptable color changes that should be considered in esthetic treatment and dietary counseling.

## 1. Introduction

Increasing esthetic demands in modern dentistry have expanded the criteria used to evaluate restorative materials. The success of restorative materials is now assessed not only according to their mechanical and physical properties but also according to their ability to provide satisfactory esthetic outcomes [1]. Among available restorative materials, composite resins are widely used because of their favorable optical properties, which allow them to mimic the appearance of natural teeth closely [2]. Ideally, composite restorations should maintain their original color while harmonizing with the surrounding dental tissues throughout their clinical service life. However, previous studies have demonstrated that the color may undergo changes over time, potentially compromising the esthetic appearance of restorations [3].

Discoloration of composite resins is a multifactorial process involving both extrinsic and intrinsic mechanisms [4]. In the oral environment, restorative materials may undergo extrinsic staining through the adsorption and absorption of pigments originating from dietary sources, beverages, plaque accumulation, smoke, and mouthrinses [5]. Intrinsic discoloration, in contrast, is associated with physicochemical changes within the material, including residual monomer oxidation, water sorption, and filler-related characteristics [2]. The extent of discoloration is further influenced by several material-dependent factors, such as resin matrix composition, degree of conversion, filler properties, photoinitiator system, and water sorption behavior [6]. Variations in these characteristics may modify the interaction between the restorative material and the oral environment, thereby affecting the long-term color stability of composite restorations.

Most studies investigating the discoloration of resin-based restorative materials have focused on conventional staining agents, such as coffee, tea, red wine, cola, and other widely consumed beverages [4,7,8]. However, evolving dietary habits and increasing health consciousness have led to a growing consumption of alternative beverages. Freshly pressed fruit juices, detox blends, and vitamin-enriched functional waters are increasingly consumed as healthier alternatives to traditional drinks [3]. Unlike conventional staining agents, these beverages may contain a diverse range of chromogenic compounds, including chlorophyll-, carotene-, and anthocyanin-derived pigments, and may differ considerably in their acidity and organic acid composition [9]. These characteristics may contribute to discoloration through both pigment adsorption and degradation-related alterations of restorative materials, potentially influencing color stability through mechanisms distinct from those associated with conventional beverages [10].

Although the staining potential of conventional beverages has been extensively documented, relatively few studies have examined the effects of these health-oriented beverages on the color stability of resin-based restorative materials [3,9,11,12,13,14]. Furthermore, information regarding the behavior of bulk-fill resin composites following exposure to such increasingly popular beverages remains limited. Bulk-fill resin composites are designed with increased translucency and modified filler systems to facilitate adequate light penetration and polymerization in single increments of up to 4–5 mm thickness [15]. These compositional modifications, including a relatively higher resin matrix content and greater inter-filler spacing, may increase their susceptibility to pigment uptake and surface degradation compared with conventional resin composites [7]. While several studies have compared the color stability of bulk-fill and conventional composites using traditional staining beverages [7,10,16], no study to date has evaluated this comparison using the newer generation of health-oriented beverages that are increasingly consumed by patients. Therefore, the aim of the present in vitro study was to evaluate and compare the color stability of a conventional nanofilled composite resin (FU) and a bulk-fill composite resin (FB) after immersion in six different solutions: distilled water (control), orange juice, green detox, red detox, pomegranate juice, and multivitamin water. The null hypotheses tested were as follows: (1) there would be no significant difference in color change (ΔL*, Δa*, Δb*, ΔE) among the six immersion solutions within each composite resin material, and (2) there would be no significant difference in color change between the conventional and bulk-fill composite resins within each immersion solution.

## 2. Materials and Methods

### 2.1. Specimen Preparation

Two commercially available resin composite materials were used in this study (Table 1): a conventional nanofilled composite (Filtek Ultimate, FU; shade A2, 3M ESPE, St. Paul, MN, USA) and a bulk-fill composite (Filtek Bulk Fill, FB; shade A2, 3M ESPE, St. Paul, MN, USA).

A power analysis (α = 0.05, power = 0.95, effect size = 0.7) using G*Power 3.1 software (Heinrich-Heine-Universität Düsseldorf, Düsseldorf, Germany) indicated that at least 10 specimens per group were required for meaningful comparisons. A total of 120 disc-shaped specimens were fabricated (n = 10 per group). Uncured composite was packed into cylindrical Teflon molds (10 mm diameter × 2 mm thickness) and pressed between two glass slides covered with Mylar strips to obtain a flat, standardized surface. Specimens were light-cured using an LED curing unit (VALO Grand; Ultradent Products, South Jordan, UT, USA), equipped with a 12-mm lens, at an irradiance of 1000 mW/cm^2^ for 20 s from each side, with a 1-mm-thick glass slide interposed between the light guide tip and the specimen surface, resulting in a standardized curing distance of 1 mm. Light intensity was verified with a radiometer (COXO; Foshan, Guangdong, China) prior to the curing procedure.

After polymerization, specimens were removed from the molds and inspected for surface defects or voids under 30× magnification. Defective specimens were discarded and replaced. All specimen preparation procedures were performed by the same operator to ensure consistency. The specimens were then stored in distilled water at 37 °C for 24 h to allow for post-cure polymerization prior to baseline color measurements. The upper and lower surfaces of each specimen were subsequently finished by sequential polishing with 600-, 800-, 1000-, and 2000-grit silicon carbide papers under water cooling. For accurate matching of baseline and final measurements, the non-measurement surface of each specimen was labeled with a unique identification number.

### 2.2. Immersion Solutions

Specimens from each composite material were randomly allocated into six groups (n = 10) according to the immersion solution using a computer-generated randomization sequence, with the operator performing the color measurements blinded to group assignments: distilled water (control), orange juice (Exotic Orange Juice; GE.TA, Adana, Türkiye), green detox juice (Juico Green Detox Blend; Dese, İstanbul, Türkiye), red detox juice (Juico Red Detox Blend; Dese, İstanbul, Türkiye), pomegranate juice (Exotic Pomegranate Juice; GE.TA, Adana, Türkiye), and multivitamin water (Saol Vitamin Water Multi-V; OHBE, İstanbul, Türkiye). The compositions and manufacturer details of the immersion solutions are presented in Table 2.

Specimens were immersed in 20 mL of their respective solutions in sealed containers and stored at 37 °C in an incubator. Solutions were renewed daily to prevent saturation and degradation. The total immersion period was 12 days [17].

### 2.3. Color Measurement

Color measurements were performed using a clinical type spectrophotometer (EasyShade 5, Vita-Zahnfabrik, Bad Säckingen, Germany) according to the CIE L*a*b* color system, where L* represents lightness (0 = black, 100 = white), a* represents the red-green axis (+a* = red, −a* = green), and b* represents the yellow–blue axis (+b* = yellow, −b* = blue). The device was calibrated according to the manufacturer’s recommendations before each measurement session. All color measurements were performed by the same operator against a white background under standard illuminant D65.

Baseline measurements (L_0_*, a_0_*, b_0_*) were recorded after the initial 24-h water storage. Final measurements (L_1_*, a_1_*, b_1_*) were obtained after the immersion period. Prior to each measurement, specimens were rinsed under running water and gently dried with filter paper. The probe tip was positioned perpendicular to the specimen surface, and three readings were taken from the center of each specimen and averaged. Color differences for each coordinate were calculated as ΔL* = L_1_* − L_0_*, Δa* = a_1_* − a_0_*, and Δb* = b_1_* − b_0_*. The overall color difference (ΔE) was calculated using the following formula:

ΔE = (ΔL*^2^ + Δa*^2^ + Δb*^2^)^1/2^

The 50:50% perceptibility and acceptability thresholds for CIELAB color differences (ΔE) were set at 1.2 and 2.7, respectively [18].

### 2.4. Statistical Analysis

All statistical analyses were performed using IBM SPSS Statistics (version 31.0; IBM Corp., Armonk, NY, USA). Descriptive statistics were calculated as mean ± standard deviation for each group. Data normality was assessed using the Shapiro–Wilk test, and homogeneity of variances was evaluated using Levene’s test.

A two-way analysis of variance (ANOVA) was conducted for each color parameter (ΔL*, Δa*, Δb*, and ΔE) with material and immersion solution as independent factors. Since Levene’s test indicated heterogeneous variances across all parameters (*p* < 0.05), the Games-Howell post hoc test was used for pairwise comparisons among immersion solutions within each material, as it does not assume equal variances or equal sample sizes. Differences between the two composite materials within each immersion solution were assessed using Welch’s *t*-test. The significance level was set at α = 0.05 for all analyses.

## 3. Results

The two-way ANOVA revealed statistically significant main effects of material and immersion solution, as well as a significant interaction between these factors, for all four color parameters (ΔL*, Δa*, Δb*, and ΔE) (*p* < 0.001). The interaction effect indicated that the influence of the immersion solution on color change depended on the type of composite resin used. Consequently, simple effects analyses were performed using Games-Howell and Welch’s *t*-test for within-material and between-material comparisons, respectively.

### 3.1. Lightness Change (ΔL*)

Mean ΔL* values and statistical groupings are presented in Table 3. In FU, distilled water and orange juice produced slight positive ΔL* shifts (0.68 ± 0.52 and 0.08 ± 0.42, respectively) that were not statistically different from each other. All other solutions caused a decrease in lightness, with red detox showing the greatest darkening (−4.75 ± 0.58).

In FB, orange juice produced a pronounced increase in lightness (16.12 ± 1.37), which was significantly higher than all other groups. In contrast, pomegranate juice caused the greatest darkening (−10.24 ± 0.28). All six immersion solutions produced significantly different ΔL* values from one another in this material.

Between-material comparisons showed that ΔL* values for FB were significantly different from FU in four of the six solutions: orange juice, green detox, red detox, and pomegranate juice (*p* < 0.01). No significant differences were found for distilled water or multivitamin water.

### 3.2. Red-Green Shift (Δa*)

Changes along the a* axis were relatively small in magnitude compared to the other coordinates (Table 3). In FU, each solution produced a statistically distinct Δa* value, ranging from a slight shift toward red with multivitamin water (0.76 ± 0.08) to a shift toward green with green detox (−1.18 ± 0.05).

In FB, multivitamin water again showed the highest positive shift (0.65 ± 0.17). Distilled water and pomegranate juice were not significantly different from each other. Orange juice and green detox shared a statistical grouping, as did orange juice and red detox.

Between-material differences reached significance for orange juice (*p* = 0.048), red detox (*p* = 0.016), and pomegranate juice (*p* = 0.004).

### 3.3. Yellow-Blue Shift (Δb*)

The Δb* values showed the widest range of variation across groups (Table 3). In FU, orange juice produced the largest positive shift toward yellow (5.10 ± 2.10), which was significantly higher than all other solutions. Distilled water, red detox, and multivitamin water formed a homogeneous group, while green detox and pomegranate juice constituted another.

In FB, red detox and multivitamin water showed the highest Δb* values (7.67 ± 0.81 and 7.20 ± 0.43, respectively) and were not significantly different from each other. Orange juice shared a grouping with multivitamin water but was significantly lower than red detox. Pomegranate juice showed a marked shift toward blue (−9.56 ± 0.47), distinctly separated from all other solutions.

Between-material comparisons revealed significant differences for all solutions except orange juice (*p* = 0.136). Notably, distilled water, which showed no significant difference between materials in the ΔL* analysis, reached significance for Δb* (*p* = 0.014).

### 3.4. Overall Color Change (ΔE)

Mean ΔE values are presented in Table 4. In FU, ΔE ranged from 1.76 ± 0.76 (distilled water) to 5.16 ± 2.08 (orange juice). Distilled water was the only solution that differed significantly from the other five, which formed overlapping statistical groups. Orange juice and red detox showed the highest ΔE values and shared a grouping.

In FB, all six solutions were significantly different from one another. Orange juice produced the highest ΔE (17.33 ± 1.61), followed by pomegranate juice (14.01 ± 0.33), red detox (10.08 ± 0.72), multivitamin water (7.48 ± 0.38), green detox (6.40 ± 0.48), and distilled water (2.68 ± 0.78).

**Figure 1:**
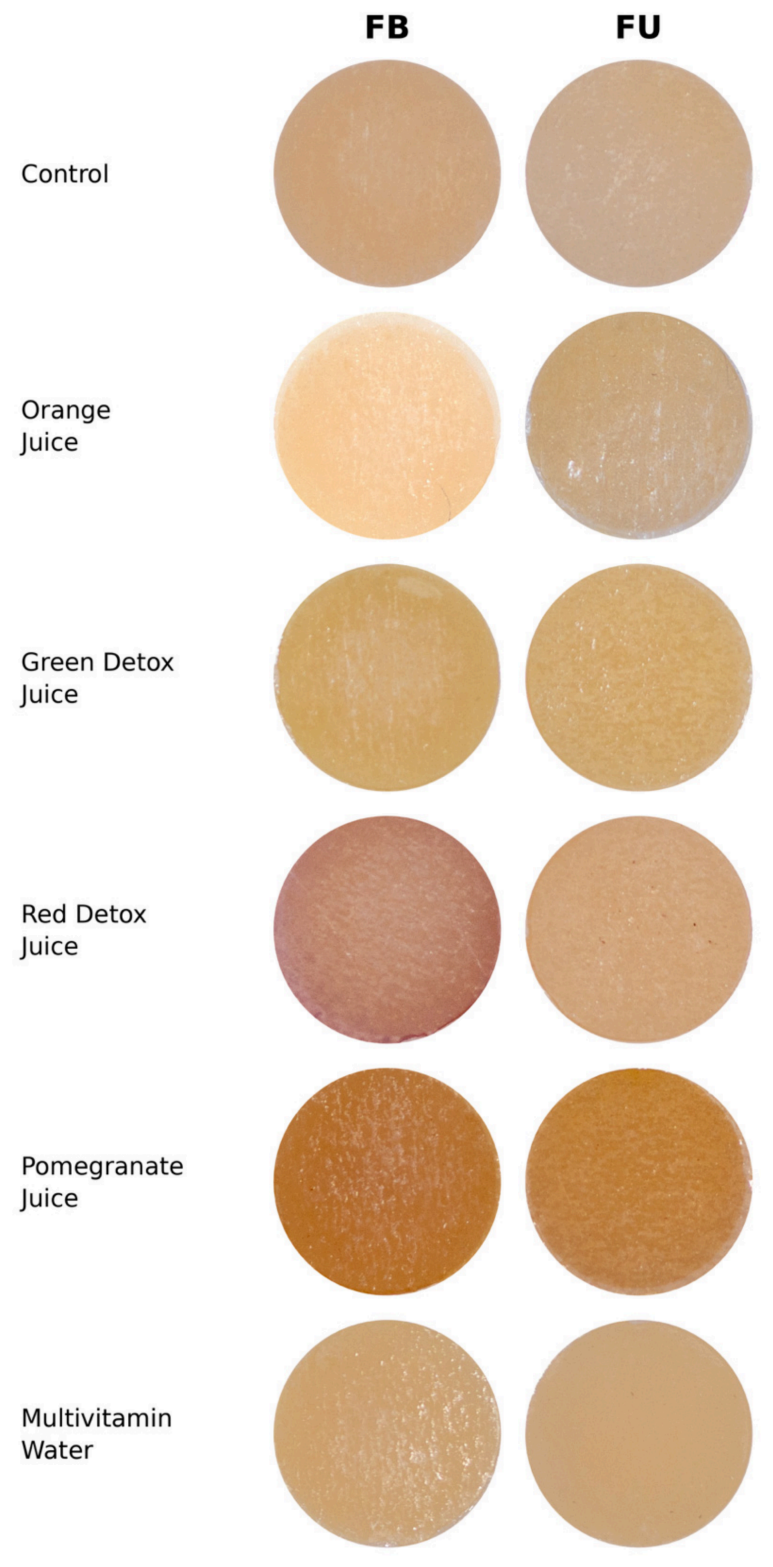
Representative images of the composite specimens after 12-day immersion in the six test solutions. Columns show the two materials, Filtek Bulk Fill (FB) and Filtek Ultimate (FU); rows show the immersion solutions: distilled water (control), orange juice, green detox juice, red detox juice, pomegranate juice, and multivitamin water. The bulk-fill composite (FB) shows visibly greater discoloration than the conventional composite (FU) across all beverages.

**Figure 2:**
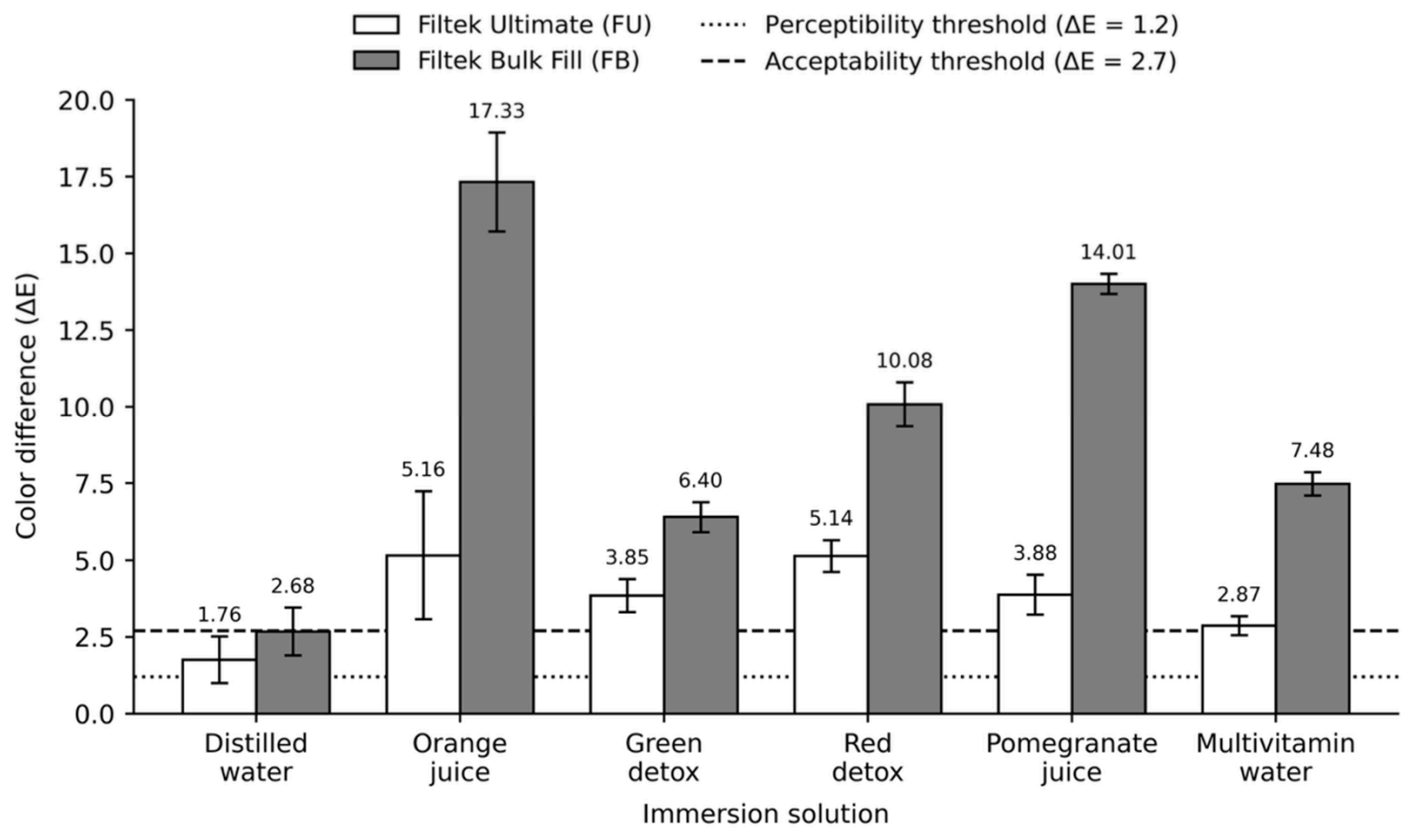
Mean color difference (ΔE) values of the two composite resins after 12-day immersion in the six test solutions. Error bars represent standard deviations (n = 10). Dotted and dashed horizontal lines indicate the 50:50% perceptibility (ΔE = 1.2) and acceptability (ΔE = 2.7) thresholds for CIELAB color differences, respectively. FU, Filtek Ultimate; FB, Filtek Bulk Fill.

FB exhibited significantly higher ΔE values than FU across all six immersion solutions, including distilled water (*p* = 0.014). The largest absolute difference between materials was observed with orange juice, where FB yielded a mean ΔE approximately 3.4 times that of FU. The visible color changes across materials and immersion solutions are shown in Figure 1, and the corresponding mean ΔE values in relation to the perceptibility and acceptability thresholds are presented in Figure 2.

According to the established perceptibility (ΔE = 1.2) and acceptability (ΔE = 2.7) thresholds for CIELAB color differences, all groups exceeded the perceptibility threshold. In FU, the distilled water group remained below the acceptability threshold (ΔE = 1.76 ± 0.76), whereas all beverage groups exceeded this limit. In FB, all beverage groups exceeded the acceptability threshold, and the distilled water group (ΔE = 2.68 ± 0.78) remained marginally below the acceptability limit.

## 4. Discussion

The present study evaluated the color stability of a conventional nanofilled composite (FU) and a bulk-fill composite (FB) following immersion in beverages associated with current health-oriented dietary trends. The results demonstrated that both the type of immersion solution and the composite material had a significant influence on color change, and that a significant interaction existed between these two factors for all color parameters tested. Accordingly, both null hypotheses were rejected. The first null hypothesis, which stated that there would be no significant difference in color change among immersion solutions within each material, was rejected because each solution produced distinct color changes; in FB, all six solutions differed significantly from one another, while in FU, distilled water was significantly different from the remaining solutions. The second null hypothesis, which stated that there would be no significant difference between the two materials, was also rejected because FB exhibited significantly greater ΔE values than FU across all six immersion solutions, including the distilled water control.

The clinical relevance of the observed changes was assessed using the perceptibility (ΔE = 1.2) and acceptability (ΔE = 2.7) thresholds for CIELAB color differences. Thus, clinical relevance was interpreted not solely on the basis of statistical differences between groups, but also according to whether the observed color changes exceeded established perceptibility and acceptability thresholds. All groups exceeded the perceptibility threshold. The distilled water controls of both materials remained within the acceptable range, although the FB control (ΔE = 2.68 ± 0.78) approached the acceptability limit, whereas all beverage groups in both materials exceeded this threshold. In FB, every beverage produced color changes well above the acceptability limit, and the magnitude of these changes was consistently greater than that observed for FU. These findings indicate that lower staining resistance, as observed for the bulk-fill material, may compromise long-term esthetic performance under frequent exposure to such beverages.

The increasing consumption of cold-pressed juices, detox beverages, and fruit-based drinks, valued for their perceived health benefits, has introduced beverages whose acids and natural colorants may adversely affect the color stability of resin-based composites [3,9,12]. Consistent with previous reports on detox, fruit-based, and acidic beverages [10,14], the present findings confirm that such drinks can induce clinically relevant discoloration. Among the tested beverages, orange juice produced the highest color change in both materials, followed by pomegranate juice and red detox juice, consistent with previous reports in which orange and pomegranate juices caused considerable discoloration and orange juice exceeded cola, both surpassing distilled water [10,13,19,20]. The staining behavior of these beverages appears closely related to their natural pigment composition (chlorophylls, carotenoids, anthocyanins, and betalains) [11]. The red detox beverage contained beetroot, carrot, apple, lemon, and ginger, providing betalains and anthocyanins previously associated with composite discoloration, which may explain its elevated ΔE values [4,5,7]. In contrast, the green detox beverage produced lower color changes than red detox and pomegranate juice despite containing multiple pigment sources, suggesting that staining potential depends more on pigment composition than on the number of pigment-containing ingredients [9,12,14].

Analysis of the individual color coordinates provided further insight into the nature of the observed discoloration. The predominantly negative ΔL* values indicated a reduction in lightness and a tendency toward darker appearances following immersion, particularly in the red detox and pomegranate juice groups, consistent with previous reports involving pigment-rich beverages [11]. Changes along the a* axis were comparatively limited, suggesting that shifts in the red–green dimension contributed less to overall discoloration than changes in lightness and yellowness/blueness. In contrast, marked alterations were observed in the Δb* coordinate, indicating that the yellow–blue axis was strongly influenced by the immersion media. Orange juice produced the greatest positive Δb* values in FU, reflecting a pronounced shift toward yellow that may be attributed to carotenoid pigments [11]. Similarly, red detox juice and multivitamin water generated substantial positive Δb* values in FB, whereas pomegranate juice caused a pronounced negative Δb* shift toward the blue region of the color space. Previous studies have shown that beverages rich in anthocyanins and betalains can induce characteristic alterations in color coordinates because of their intense natural pigmentation [11,21]. Overall, the greater variations observed in Δb* compared with Δa* suggest that beverage pigments predominantly affected the yellow–blue component of color, highlighting the important role of pigment type and concentration in determining the direction and magnitude of color change in resin composite materials [11,21].

A particularly distinctive finding was the pronounced positive ΔL* shift observed for orange juice in FB, which increased markedly in lightness rather than darkening as observed with the other beverages. Color changes in resin composites may arise from different mechanisms, including the uptake or adsorption of beverage pigments and chemical or physical alterations of the resin matrix induced by acidic exposure. In the present study, the pronounced increase in lightness observed for orange juice in FB may reflect surface-related optical changes associated with its acidic environment in addition to pigment uptake. Because both pigment uptake and acid-related surface or matrix alterations may occur simultaneously during beverage exposure, the relative contribution of these mechanisms to the observed color changes could not be determined in the present study. Orange juice contains predominantly carotenoid pigments with limited direct staining capacity, but also has a low pH and high citric acid content; therefore, this response may be partly related to acid-related surface changes in the composite surface. Organic acids have been shown to soften the resin matrix and potentially promote surface erosion, with the degree of softening being strongly dependent on acidity [22]. Such surface alterations may increase surface irregularity, thereby affecting light scattering and surface reflection and consequently influencing the optical behavior of resin composites. These changes may have contributed to the higher ΔL* values observed in the present study [23,24]. The more translucent structure and lower filler loading of FB may have rendered it more susceptible to such acid-related effects than FU, consistent with the pronounced ΔL* increase observed only in the bulk-fill material. A comparable mechanism may partly account for the relatively high color change recorded for multivitamin water in FB, which produced a marked positive Δb* shift despite its limited pigment content. This beverage contains citric acid as an acidity regulator together with fruit juice concentrates, and its acidic nature may have contributed to surface alterations and consequent color change beyond what its pigment content alone would suggest. Nevertheless, these explanations remain speculative because surface roughness or other surface characteristics were not directly assessed in the present study. In particular, no surface roughness measurements using profilometry or atomic force microscopy (AFM) were performed; therefore, acid-induced surface degradation or matrix erosion could not be directly confirmed. Future studies incorporating quantitative surface characterization are warranted to test this proposed mechanism.

Material-related factors may also explain the significantly higher discoloration observed in FB compared with FU. The staining susceptibility of resin-based composites is strongly influenced by the composition of the resin matrix, as water sorption occurs primarily within this phase and facilitates the uptake of staining agents [4,5,7,12]. Increased water sorption may soften the resin matrix and enhance discoloration [5,12]. Previous studies have demonstrated that hydrophilic monomers facilitate fluid uptake into the resin matrix, and that water sorption generally follows the order TEGDMA > Bis-GMA > UDMA > Bis-EMA; consequently, materials richer in UDMA or Bis-EMA tend to exhibit lower water sorption and greater resistance to discoloration [4,7,12,25]. In the present study, FU contains Bis-GMA, UDMA, TEGDMA, PEGDMA, and Bis-EMA, whereas FB contains AUDMA, UDMA, and 1,12-dodecane-DMA. Therefore, differences in monomer composition and the resulting water sorption behavior may have contributed to the distinct staining responses observed between the two materials. The presence of UDMA and Bis-EMA in FU may partly be associated with its superior color stability; however, because the materials differ in several compositional aspects simultaneously and polymer network characteristics and degree of conversion were not directly characterized in the present study, the contribution of individual monomers or polymer network structure to the observed discoloration cannot be determined. These interpretations should therefore be considered as possible explanations rather than directly confirmed mechanisms. Beyond facilitating discoloration, water sorption may also promote microcrack formation and hydrolytic degradation that enhance stain penetration [5]. In addition, oxidation of residual unreacted monomers can contribute to intrinsic color change, and since a higher degree of conversion reduces residual monomer content, the lower degree of conversion reported for some bulk-fill composites may partly explain the greater discoloration of FB [2,5,6,15,26]. Because the degree of conversion was not measured here, this explanation remains tentative and should not be interpreted as a confirmed mechanism.

Filler characteristics may also contribute to the staining susceptibility of resin-based composites. A higher filler loading reduces the proportion of resin matrix within the material, thereby limiting water sorption and the uptake of staining agents, which may ultimately improve color stability [4]. Consistent with this mechanism, increased resin matrix content has been associated with greater discoloration, whereas a higher filler ratio has been reported to reduce color change [4,5]. In the present study, FU exhibited a slightly higher filler loading than FB (78.5 wt% and 63.5 vol% versus 76.5 wt% and 58.4 vol%, respectively), which may have reduced the amount of resin matrix available for water uptake and stain absorption. In addition to filler loading, filler particle size may influence discoloration behavior. Larger filler particles have been reported to be more susceptible to color change during water storage because of hydrolytic degradation at the filler–matrix interface [12]. In contrast, smaller filler particles are generally associated with smoother surfaces and reduced extrinsic staining [7,27]. Supporting this view, lower color change values have been reported for nanofilled composites compared with hybrid composites [28,29]. Therefore, the nanofilled structure and higher filler loading of FU may have contributed to its lower ΔE values compared with FB. Nevertheless, because filler characteristics and resin matrix composition interact simultaneously, the independent contribution of each factor to the observed discoloration behavior cannot be determined from the present findings alone. Importantly, the present findings are specific to the tested FB formulation and should not be generalized to bulk-fill composites as a material category. Differences in resin chemistry, filler composition, filler loading, and polymerization characteristics among bulk-fill products may result in different color stability profiles. Therefore, comparative studies involving multiple bulk-fill brands and formulations are warranted to determine whether the observed pattern is consistent across this material category.

Overall, these findings indicate that both material- and beverage-related factors influence the long-term esthetic performance of resin composite restorations. From a clinical perspective, color stability should be considered when selecting restorative materials for patients who frequently consume highly pigmented or acidic beverages, and such patients should be informed of the associated risk of restoration discoloration. The immersion duration was selected based on previously published artificial-aging protocols for resin composites. In particular, Biçer et al. used a 12-day immersion period to evaluate the color stability of resin composites in different immersion solutions [17]. These results should nonetheless be interpreted within the limitations of the in vitro design and the continuous 12-day static immersion protocol, which does not reproduce the dynamic conditions of the oral environment. In particular, the present model did not account for salivary buffering and dilution, enzymatic activity, pH fluctuations, temperature variation and thermal cycling, or mechanical abrasion associated with toothbrushing and mastication, all of which may modify the color behavior of resin composites in vivo. Saliva may reduce the contact time and concentration of dietary pigments and acids through dilution and buffering, potentially attenuating the color changes observed under continuous immersion conditions. Accordingly, the continuous static exposure used in the present study may not fully reflect the intermittent and dynamic pattern of beverage exposure that occurs clinically. In addition, color measurements were performed over a white background only; therefore, changes in opacity or translucency were not directly assessed. Future studies should therefore employ more clinically representative aging protocols, including simulated salivary conditions, thermal cycling, and chewing and toothbrushing simulation. Color measurements over both white and black backgrounds would also allow translucency changes to be assessed. Finally, quantitative surface characterization, such as microhardness and surface roughness, together with determination of the degree of conversion by FTIR, would help clarify the mechanisms underlying the observed discoloration.

## 5. Conclusions

Within the limitations of this in vitro study, the bulk-fill composite (FB) was significantly more susceptible to discoloration than the conventional nanofilled composite (FU) for all tested beverages. Orange juice produced the highest color change in both materials and was associated with a pronounced increase in lightness in the bulk-fill composite, which may be related to acid-induced surface changes in addition to pigment absorption. However, because surface roughness and other surface characteristics were not measured, this proposed mechanism could not be directly confirmed. Pomegranate juice also produced marked color change, characterized by darkening and a shift toward the blue region. All health-oriented beverages, including detox juices and multivitamin water, produced color changes exceeding the acceptability threshold in both materials, but the magnitude of these changes was substantially greater in the bulk-fill composite. Clinicians should therefore consider the discoloration potential of these increasingly consumed beverages when selecting bulk-fill composites for esthetic regions and counsel patients accordingly.

## Figures and Tables

**Table 1:** Composites used in the study.

Brand	Manufacturer	Chemical Composition	Classification
Filtek Ultimate (FU)	3M, St. Paul, MN, USA	Organic matrix: Bis-GMA, UDMA, TEGDMA, PEGDMA, Bis-EMA. Filler (particle size): Silica (20 nm), zirconia (4 to 11 nm), zirconia/silica cluster (0.6–10 micron) Filler weight/Volume: 78.5%/63.5%	Nanofilled
Filtek Bulk Fill (FB)	3M, St. Paul, MN, USA	Organic matrix: AUDMA, UDMA and 1, 12-dodecane-DMA Filler (particle size): YbF3 (100 nm), silica (20 nm), zirconia (4 to 11 nm) Filler weight/Volume: 76.5 (wt), 58.4 (vol)	High-viscosity bulk fill

Bis-GMA, bisphenol-glycidyl methacrylate; UDMA, urethane dimethacrylate; TEGDMA, triethyleneglycol dimethacrylate; PEGDMA, polyethylene glycol dimethacrylate; Bis-EMA, ethoxylated bisphenol-A dimethacrylate; AUDMA, aromatic urethane dimethacrylate; DMA, dimetachrylate; YbF3, ytterbium trifluoride.

**Table 2:** Immersion solutions used in the study.

Material/Brand	Manufacturer	Ingredients
Juico Red Detox Blend	Dese, İstanbul, Türkiye	Apple, lemon, ginger, carrot, beetroot. Preservative-free, additive-free, colorant-free, sugar-free.
Juico Green Detox Blend	Dese, İstanbul, Türkiye	Apple, spinach, lemon, kale, lettuce, parsley, cucumber, ginger. Preservative-free, additive-free, colorant-free, sugar-free.
Exotic Orange Juice	GE.TA, Adana, Türkiye	Orange juice. Preservative-free, colorant-free, contains sugar.
Exotic Pomegranate Juice	GE.TA, Adana, Türkiye	Pomegranate juice. Preservative-free, colorant-free, contains sugar.
Saol Vitamin Water Multi-V (Multivitamin Water)	OHBE, İstanbul, Türkiye	Water, sugar, orange and apple juice concentrates (minimum 0.73%), thickener (pectin), acidity regulator (citric acid), peach flavoring, sweetener (steviol glycosides), natural plant and vegetable extract, vitamins A, C (ascorbic acid), E, D, niacin, vitamin B6, biotin, vitamin B12, and zinc mineral.

**Table 3:** Mean ± standard deviations of color coordinates (ΔL*, Δa*, Δb*) for each composite resin and immersion solution.

Color Coordinate		Material		Difference
	Solution	FU	FB	*p* Value
ΔL*	Distilled water	0.68 ± 0.52 ^a^	0.96 ± 0.68 ^b^	0.324
	Orange juice	0.08 ± 0.42 ^a^	16.12 ± 1.37 ^a^	<0.001 ***
	Green detox	−3.61 ± 0.56 ^c^	−4.57 ± 0.57 ^d^	0.001 **
	Red detox	−4.75 ± 0.58 ^d^	−6.47 ± 0.64 ^e^	<0.001 ***
	Pomegranate juice	−3.79 ± 0.63 ^c^	−10.24 ± 0.28 ^f^	<0.001 ***
	Multivitamin water	−2.08 ± 0.53 ^b^	−1.87 ± 0.39 ^c^	0.338
Δa*	Distilled water	−0.01 ± 0.11 ^b^	0.10 ± 0.31 ^b^	0.294
	Orange juice	−0.66 ± 0.07 ^d^	−0.96 ± 0.41 ^cd^	0.048 *
	Green detox	−1.18 ± 0.05 ^f^	−1.08 ± 0.15 ^d^	0.063
	Red detox	−0.82 ± 0.07 ^e^	−0.55 ± 0.29 ^c^	0.016 *
	Pomegranate juice	−0.44 ± 0.07 ^c^	−0.16 ± 0.24 ^b^	0.004 **
	Multivitamin water	0.76 ± 0.08 ^a^	0.65 ± 0.17 ^a^	0.098
Δb*	Distilled water	1.56 ± 0.71 ^b^	2.42 ± 0.70 ^d^	0.014 *
	Orange juice	5.10 ± 2.10 ^a^	6.27 ± 1.04 ^b^	0.136
	Green detox	0.30 ± 0.58 ^c^	4.17 ± 1.29 ^c^	<0.001 ***
	Red detox	1.68 ± 0.56 ^b^	7.67 ± 0.81 ^a^	<0.001 ***
	Pomegranate juice	−0.40 ± 0.59 ^c^	−9.56 ± 0.47 ^e^	<0.001 ***
	Multivitamin water	1.74 ± 0.40 ^b^	7.20 ± 0.43 ^ab^	<0.001 ***

Abbreviations: FU, Filtek Ultimate; FB, Filtek Bulk Fill. Different lower-case superscript letters indicate significant differences among immersion solutions within each material (Games-Howell test, *p* < 0.05). Differences between materials for each solution were assessed using Welch’s *t*-test. *, *p* < 0.05; **, *p* < 0.01; ***, *p* < 0.001.

**Table 4:** Mean ± standard deviation of color differences (ΔE) for each composite resin and immersion solution.

Material	Solution	ΔE
Filtek Ultimate	Distilled water	1.76 ± 0.76 ^d,B^
	Orange juice	5.16 ± 2.08 ^abc,B^
	Green detox	3.85 ± 0.54 ^b,B^
	Red detox	5.14 ± 0.52 ^a,B^
	Pomegranate juice	3.88 ± 0.65 ^b,B^
	Multivitamin water	2.87 ± 0.31 ^c,B^
Filtek Bulk Fill	Distilled water	2.68 ± 0.78 ^f,A^
	Orange juice	17.33 ± 1.61 ^a,A^
	Green detox	6.40 ± 0.48 ^e,A^
	Red detox	10.08 ± 0.72 ^c,A^
	Pomegranate juice	14.01 ± 0.33 ^b,A^
	Multivitamin water	7.48 ± 0.38 ^d,A^

Different lower-case superscript letters indicate significant differences among immersion solutions within each material (Games-Howell test), whereas different upper-case letters indicate significant differences between composite resins (Welch’s *t*-test) (*p* < 0.05).

## Data Availability

The data presented in this study are available on request from the corresponding author due to the Datasets Generated during the Current Study are not Publicly Available due to Their Use in Ongoing Research and Potential Future Publications but are Available from the Corresponding Author on Reasonable Request.
